# Cellular defects caused by hypomorphic variants of the Bloom syndrome helicase gene *BLM*

**DOI:** 10.1002/mgg3.188

**Published:** 2015-11-26

**Authors:** Vivek M. Shastri, Kristina H. Schmidt

**Affiliations:** ^1^ Department of Cell Biology, Microbiology and Molecular BiologyUniversity of South FloridaTampaFlorida33620; ^2^ Graduate Program in Cell and Molecular BiologyUniversity of South FloridaTampaFlorida33620; ^3^ Cancer Biology and Evolution ProgramH. Lee Moffitt Cancer Center and Research InstituteTampaFlorida33612

**Keywords:** BLM variants, bloom syndrome, DNA helicase, genome instability, hypomorphic

## Abstract

**Background:**

Bloom syndrome is an autosomal recessive disorder characterized by extraordinary cancer incidence early in life and an average life expectancy of ~27 years. Premature stop codons in *BLM*, which encodes a DNA helicase that functions in DNA double‐strand‐break repair, make up the vast majority of Bloom syndrome mutations, with only 13 single amino acid changes identified in the syndrome. Sequencing projects have identified nearly one hundred single nucleotide variants in *BLM* that cause amino acid changes of uncertain significance.

**Methods and Results:**

Here, in addition to identifying five BLM variants incapable of complementing certain defects of Bloom syndrome cells, making them candidates for new Bloom syndrome causing mutations, we characterize a new class of BLM variants that cause some, but not all, cellular defects of Bloom syndrome. We find elevated sister‐chromatid exchanges, a delayed DNA damage response and inefficient DNA repair. Conversely, hydroxyurea sensitivity and quadriradial chromosome accumulation, both characteristic of Bloom syndrome cells, are absent. These intermediate variants affect sites in BLM that function in ATP hydrolysis and in contacting double‐stranded DNA.

**Conclusion:**

Allele frequency and cellular defects suggest candidates for new Bloom syndrome causing mutations, and intermediate BLM variants that are hypomorphic which, instead of causing Bloom syndrome, may increase a person's risk for cancer or possibly other Bloom‐syndrome‐associated disorders, such as type‐2 diabetes.

## Introduction

Bloom syndrome (MIM 210900 for *BLM*) is a rare chromosome breakage disorder characterized by short stature and an extraordinary predisposition to a broad range of cancers, often multiple, early in life (German and Passarge 1989; German and Ellis 1998). Other symptoms that can be present in persons with Bloom syndrome include sun‐sensitive skin with telangiectatic and hyper‐ and hypo‐pigmented areas, immunodeficiency, subfertility in females and infertility in males, and type 2 diabetes mellitus (Bloom 1954; German and Passarge 1989; German and Ellis 1998; Ellis et al. 2008).

The gene defective in persons with Bloom syndrome – the tumor suppressor gene *BLM* – encodes a 3′ to 5′ DNA helicase that belongs to the evolutionarily conserved RecQ helicase family (Ellis et al. 1995; Bennett and Keck 2004). The helicase core of BLM spans amino acid residues 658 to 1197 and consists of the DNA‐dependent ATPase (DEAH) domain with seven conserved helicase motifs, and the RecQ‐C‐terminal (RQC) domain with Zn‐binding (Zn) and winged‐helix (WH) subdomains (Hickson 2003; Bennett and Keck 2004). C‐terminal of the RQC domain is the conserved Helicase and RNase D C‐terminal (HRDC) domain, which plays a role in DNA binding and is thought to regulate helicase activity (Huber et al. 2006; Kim and Choi 2010). The best understood roles for BLM are in the repair of DNA double strand breaks (DSBs) by homologous recombination (HR) where – in a complex with topoisomerase Topo III*α* and Rmi1/Rmi2 – BLM dissolves double Holliday junctions (dHJ) into noncrossover products (Hickson 2003). BLM/Topo III*α*/Rmi1/Rmi2 has also been implicated in the processing of the ends of DSBs into single‐strand 3′ overhangs for the strand invasion step of HR and in the control of HR levels by reversing strand invasion events (Bachrati et al. 2006; Daley et al. 2014; Sturzenegger et al. 2014). As a result, cells that lack functional BLM exhibit elevated levels of mitotic recombination, chromatid breaks, increased crossover formation between sister chromatids (~10‐fold higher in cells from persons with Bloom syndrome), and signs of illegitimate interchromosomal recombination events suggested by the presence of quadriradial chromosomes (Chaganti et al. 1974; Groden et al. 1990; Lonn et al. 1990; Groden and German 1992). Consistent with the role of BLM in maintaining genome integrity, Bloom syndrome cells are sensitive to genotoxic agents, including hydroxyurea (HU), which causes replication stress by depleting the nucleotide pool, and camptothecin (CPT), a natural product that interferes with replication and transcription by trapping topoisomerase I on nicked DNA. The elevated level of mitotic recombination between homologous chromosomes is thought be a major contributor to the extraordinary cancer predisposition in Bloom syndrome as it can lead to the loss of heterozygosity (LOH) at loci carrying tumor‐suppressor genes (Chester et al. 1998; Traverso et al. 2003).

**Figure 1:**
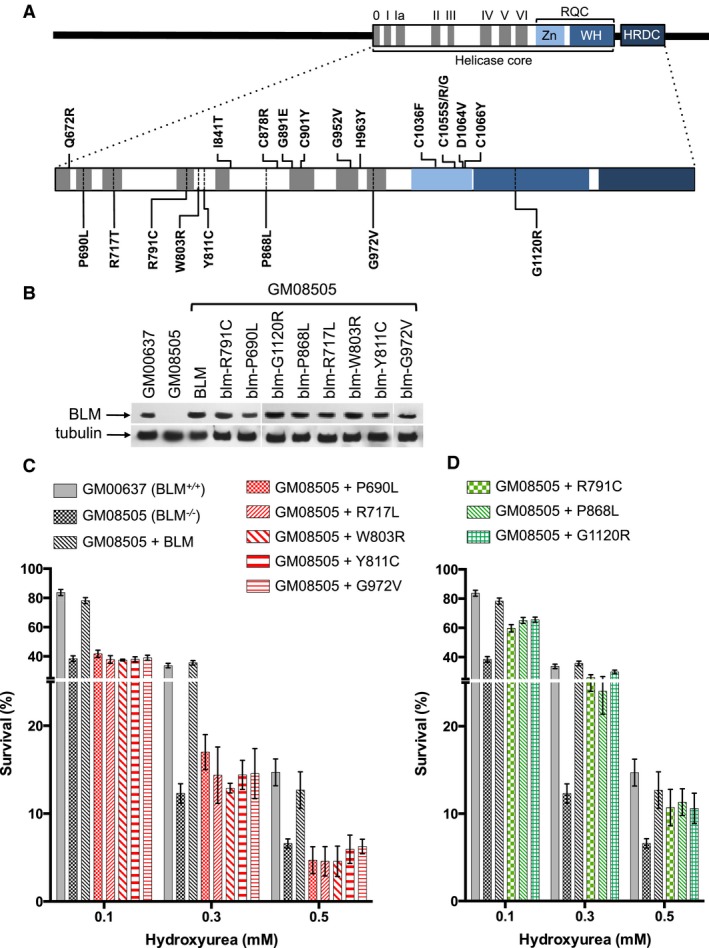
Ability of BLM variants to complement the hydroxyurea sensitivity of the BLM‐deficient cell line GM08505. (A) Map of human BLM with conserved helicase motifs I‐VI, the RecQ‐C‐terminal (RQC) domain consisting of Zn‐binding and winged helix (WH) subdomains, and the Helicase‐ and RNaseD‐C‐terminal (HRDC) domain. Sites of known Bloom syndrome causing missense mutations are indicated above the map and locations of BLM variants analyzed in this study are indicated below the map. (B) GM08505 cells, which are homozygous for the *blm* ^*ASH*^ mutation and do not express full‐length BLM (lane 2), express stably transfected BLM variants (lanes 4–11) at similar levels as wildtype BLM (lane 3). Endogenous BLM expression in the normal fibroblast cell line GM00637 is shown lane 1. (C–D) Cells were exposed to increasing concentrations of hydroxyurea (0.1–0.5 mmol/L) for 48 h and survivors (colonies with ~50 or more cells) were counted after up to 4 weeks of growth in hydroxyurea‐free media. Three independently generated stable cell lines were analyzed for BLM and every BLM variant. Mean ± SD is shown.

*BLM* is transcribed as a 97.93 kb pre‐messenger RNA, with 21 exons coding for a 1417 amino acid protein. In the majority of persons with Bloom syndrome the *BLM* gene is inactivated by small insertion/deletion mutations or nonsense mutations that lead to a premature stop codon upstream or within exons 7–18, which code for the helicase core of BLM. The most common Bloom syndrome mutation is a 6 bp deletion/7 bp insertion in exon 9 (6‐BP DEL/7‐BP INS, rs113993962:ATCTGA>TAGATTC) (Ellis et al. 1994, 1998; Li et al. 1998; Straughen et al. 1998; German et al. 2007). This frameshift indel mutation changes the amino acids encoded by codons 736–739 before causing a premature stop in codon 740 (p.Tyr736fsX4). This mutation, also referred to as *blm* ^*ASH*^, occurs with a frequency of ~0.01 in the Ashkenazi Jewish population (Li et al. 1998). In addition to mutations causing premature chain termination, 13 missense mutations in the ATPase and RQC domains of the BLM helicase core have been identified in persons with Bloom syndrome (Ellis et al. 1995; Foucault et al. 1997; Barakat et al. 2000; German et al. 2007). Seven of the missense mutations alter amino acid residues in the ATPase domain (Q672R, I841T, C878R, G891E, C901Y, G952V, H963Y) whereas the six missense mutations in the RQC domain mostly affect the highly conserved cysteine residues involved in Zn coordination (C1036F, C1055S, C1055R, C1055G, D1064V, C1066Y) (Fig. 1A). The lack of differences in the expression of Bloom syndrome between individuals with these missense mutations, either in the homozygous or compound heterozygous state, and individuals with early chain‐terminating mutations suggests that they all inactivate BLM (Ellis et al. 1995).

There is some evidence that heterozygosity for *BLM* mutations leads to increased colorectal cancer risk in humans and mice (Goss et al. 2002; Gruber et al. 2002), and causes increased sensitivity to DNA‐damaging agents in a diploid yeast model (Mirzaei and Schmidt 2012). However, it is unclear if besides fully inactivating mutations that cause Bloom syndrome other naturally occurring *BLM* mutations cause more subtle functional defects that might be new cancer risk factors in otherwise healthy individuals. To address this question we used a yeast model to screen coding single nucleotide polymorphisms (SNPs) in the human *BLM* gene for those that impair BLM function as estimated by the hypersensitivity of cells to HU (Mirzaei and Schmidt 2012). This yeast model expressed a chimera of the N‐terminal 648 residues of Sgs1 (the BLM‐related RecQ helicase in *Saccharomyces cerevisiae*) and the C‐terminal 800 residues of human BLM to which 41 nonsynonymous SNPs from the Short Genetic Variations database (dbSNP) map. We ranked these 41 coding SNPs and two additional SNPs reported in the literature (German et al. 2007) based on their probability to impair BLM function using PolyPhen (Ramensky et al. 2002) and evaluated the 22 SNPs predicted to be ‘probably’ or ‘possibly’ damaging (PolyPhen score >1.5) for their effect on BLM function in vivo (Mirzaei and Schmidt 2012). The screen identified eight SNPs that impaired BLM function, all of which had a PolyPhen score >2 (rs761589072:C>T, P690L; rs28406486:G>C, R717T; rs55880859:C>T, R791C; rs148394770:T>C, W803R; rs145029382:A>G, Y811C; rs2227935:C>T, P868L; rs150475674, G972V:G>T; rs139773499:G>A, G1120R) (Table 1) (Fig. 1A). Interestingly, this screen suggested that in addition to rare null alleles that had not yet been associated with Bloom syndrome more common *BLM* alleles may also be functionally impaired. The higher frequency of these alleles in the human population (e.g. rs2227935:C>T, P868L; 5.13%) suggests that they are insufficient for full‐scale Bloom syndrome, but their lower functional activity may lead to an increased cancer risk later in life or an increased risk for developing other symptoms of Bloom syndrome, such as type 2 diabetes mellitus or fertility problems. Here, we have quantified functional defects of cells expressing these new *BLM* alleles, with an emphasis on the first three hypomorphic *BLM* allele candidates (rs2227935, rs55880859, rs139773499) by assessing chromosomal abnormalities, their ability to respond to genotoxic agents and their ability to repair DSBs.

**Table 1:** *BLM* gene variants evaluated in this study

Nucleotide change^a^	Amino acid change^b^	dbSNP ID	Allele frequency^c^	Prediction of functional impact			
				PolyPhen^d^	PolyPhen2^e^	SIFT^f^	FIS^g^
c.2069C>T	p.Pro690Leu	rs761589072	n.a.	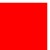 2.724	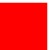 1	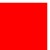 0	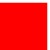 4.875
c.2150G>C	p.Arg717Thr	rs28406486	n.a.	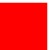 2.008	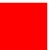 0.985	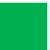 0.15	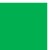 0.85
c.2371C>T	p.Arg791Cys	rs55880859	0.0002	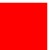 2.495	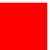 0.981	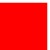 0.01	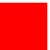 2.81
c.2407T>C	p.Trp803Arg	rs148394770	n.a.	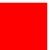 3.757	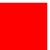 1	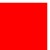 0	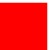 4.965
c.2432A>G	p.Tyr811Cys	rs145029382	n.a.	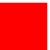 2.616	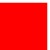 1	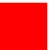 0	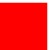 4.955
c.2603C>T	p.Pro868Leu	rs2227935	0.0513	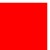 2.724	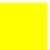 0.729	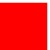 0.02	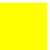 2.395
c.2915G>T	p.Gly972Val	rs367543034	n.a.	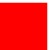 2.397	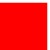 1	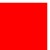 0	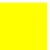 2.295
c.3358G>A	p.Gly1120Arg	rs139773499	n.a.	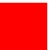 2.172	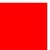 0.999	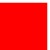 0	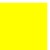 3.285

a GenBank RefSeq: NM_000057.3.

b GenBank RefSeq: NP_000048.1.

c Minor allele frequencies from dbSNP (http://www.ncbi.nlm.nih.gov/SNP/).

d Prediction using PolyPhen (http://genetics.bwh.harvard.edu/pph/index.html): red, ‘probably damaging’.

e Prediction using PolyPhen‐2 (http://genetics.bwh.harvard.edu/pph/): red, probably damaging; yellow, possibly damaging.

f Prediction using SIFT (http://sift.jcvi.org/): red, damaging; green, tolerated.

g Predicted Functional Impact Score via Mutation Assessor (http://mutationassessor.org/); red, high functional impact; yellow, medium functional impact; green, low functional impact.

## Materials and Methods

### Cell lines, plasmids, and transfection

GM08505 is an SV40‐transformed skin fibroblast cell line established from a patient with Bloom syndrome (Ellis et al. 1995) and was obtained from Coriell Cell Repository. GM00637 is an SV40‐transformed skin fibroblast cells line from an unaffected individual (Coriell Cell Repository). Cells were grown in minimal essential medium (Corning, Tewksbury, MA) supplemented with 10% FBS and 2 mmol/L glutamine at 37°C in the presence of 5% CO_2_. GM08505 cells were plated 24 h before transfections at approximately 2 × 10^4^ per cm^2^. BLM cDNA cloned into pcDNA3 vector and mutated at stated sites using site‐directed mutagenesis was transfected using Polyfect (Qiagen, Valencia, CA). Stable clones were selected in the presence of G418 (750 *μ*g/mL). Clones were maintained in the presence of G418 (350 *μ*g/mL). BLM cDNA in pcDNA3 was a kind gift from Dr. Ian Hickson, University of Copenhagen. Cell lines expressing the BLM variants evaluated in this study are listed in Table 2.

**Table 2:** Summary of functional evaluation of *BLM* alleles

*BLM* allele	dbSNP ID	Cell line^c^	HU sensitivity^d^	Chromosome abnormalities^a^		DNA damage response^b^		DNA in comet tail (%)^e^
				SCEs	Quadriradials	γH2AX accumulation	γH2AX elimination	
wildtype	n.a.	KSVS1400	−	0.18 ± 0.05	0.27 ± 0.12	−	−	67 ± 1.1/10 ± 0.3
*blm‐Ash* f	rs113993962	GM08505	+++	1.44 ± 0.05	1.33 ± 0.16	+++	+++	70 ± 0.7/32 ± 0.9
*blm‐P690L*	rs761589072	KSVS1409	+++	1.35 ± 0.07	0.91 ± 0.13	+++	+++	n.d.
*blm‐R717L*	rs28406486	KSVS1405	+++	n.d.	n.d.	n.d.	n.d.	n.d.
*blm‐R791C*	rs55880859	KSVS1425	+/−	0.74 ± 0.04	0.19 ± 0.09	+	+++	n.d.
*blm‐W803R*	rs148394770	KSVS1424	+++	1.51 ± 0.24	0.69 ± 012	n.d.	n.d.	n.d.
*blm‐Y811C*	rs145029382	KSVS1416	+++	n.d.	n.d.	n.d.	n.d.	n.d.
*blm‐P868L*	rs2227935	KSVS1419	+/−	0.81 ± 0.12	0.13 ± 0.07	+	+++	63 ± 0.7/19 ± 0.4
*blm‐G972V*	rs367543034	KSVS1413	+++	n.d.	n.d.	n.d.	n.d.	n.d.
*blm‐G1120R*	rs139773499	KSVS1410	+/−	0.94 ± 0.11	0.182 ± 0.08	+	+++	n.d.

a Sister‐chromatid exchanges (SCEs) are shown as SCEs per chromosome with standard deviation; quadriradials are shown as number of quadriradial chromosomes per metaphase (46 chromosomes) with standard deviation; n.d., not determined.

b DNA damage response was assessed by H2AX phosphorylation status; + and +++ denote mild and severe delays in the accumulation or elimination of phosphorylated H2AX after treatment with 1 *µ*mol/L CPT.

c Cell lines were generated by stable transfection of cell line GM08505 (*BLM* ^*Ash*^ */* ^*Ash*^) with pcDNA3 containing *BLM* cDNA, or *BLM* cDNA with the indicated nucleotide change. Skin fibroblast cell line GM08505 derived from an individual with Bloom syndrome was obtained from Coriell Biorepository.

d +++ more sensitive to HU than wildtype BLM complemented cells at all tested HU concentrations (0.1–0.5 mmol/L); +/− more sensitive than wildtype BLM complemented cells at low HU concentrations, but not at higher HU concentration.

e DNA double‐strand breaks were visualized by neutral comet assay; fraction of DNA (%) in the comet tail was quantified 1/48 h after release from treatment with 1 *µ*mol/L CPT; n.d. not determined.

f NM_000057.3(BLM):c.2207_2212delATCTGAinsTAGATTC (p.Tyr736Leufs).

### Subcellular fractionation and western blotting

Nuclear extracts were prepared from exponentially growing cells to detect BLM expression. Cells were lysed in 20 mmol/L Tris pH 7.4, 10 mmol/L KCl, 1 *μ*mol/L EDTA, 0.2% NP40, 50% glycerol, 0.6 mmol/L *β*‐mercaptoethanol, 1 mmol/L PMSF, and protease inhibitor cocktail (Pierce, Rockford, IL), followed by nuclear extraction in 20 mmol/L Tris pH 7.4, 10 mmol/L KCl, 0.4 mol/L NaCl, 1 mmol/L EDTA, 50% glycerol, 0.6 mmol/L BME, 1 mmol/L PMSF, and protease inhibitor cocktail (Pierce). Mouse monoclonal antibody BLM‐F5 (SCBT, Dallas, TX) was used to detect BLM and a monoclonal tubulin antibody (Abcam, Cambridge, MA) was used as a loading control.

### Differential sister‐chromatid staining

Sister chromatid exchanges (SCEs) were quantified using protocols described previously (Perry and Wolff 1974; Bayani and Squire 2001; Campos et al. 2009). Briefly, cells were cultured for two cell cycles in growth medium supplemented with BrdU Labeling Reagent (Life Technologies, Carlsbad, CA) and metaphase arrest was induced by the addition of 0.1 *μ*g/mL colcemid for 1 h. Chromosome spreads were prepared after hypotonic treatment with 75 mmol/L KCl and fixation with 3:1 (vol/vol) methanol‐acetic acid. Metaphase spreads were differentially stained using Hoechst 33258 (75 *μ*g/mL; Life Technologies) and Giemsa (3.5%; Life Technologies). Images of intact metaphases were acquired on a Zeiss Axiovert 100 deconvolution microscope with a Plan‐Apochromat 100x/1.40 Oil DIC M27 objective Zeiss, Thornwood, NY. For each cell line, a minimum of 1500 chromosomes were analyzed. The mean ± SD is reported.

### Clonogenic survival assay

Cells were seeded at a density of 500 cells/well for colony formation. On the next day, either fresh medium containing HU (0.1, 0.2, 0.3, 0.4, or 0.5 mmol/L) or drug‐free medium was added. The medium was removed after 48 h and cells were washed with PBS before colonies were allowed to grow in fresh complete growth medium for up to 4 weeks. Colonies were fixed in 3:1 (vol/vol) methanol‐acetic acid and stained with 0.01% crystal violet. Colonies with more than 50 cells were scored as survivors.

### *γ*H2AX accumulation and elimination

Exponentially growing cells were exposed to 1 *μ*mol/L CPT for 1 h, released into fresh media, and harvested at various time points (10 min, 20 min, 30 min, 45 min, 1 h, 8 h, 24 h, and 48 h). To detect *γ*H2AX, chromatin fractions were prepared from nuclear extracts using 0.5 mol/L HCl, 50% glycerol, 100 mmol/L *β*‐mercaptoethanol and then neutralized using NaOH in 40 mmol/L Tris pH 7.4 with protease inhibitor cocktail (Pierce) (Rios‐Doria et al. 2009). Chromatin fractions were separated on 16% Tricine‐SDS polyacrylamide (Schagger 2006) and Western blots were probed with PhosphoDetect Anti‐H2AX [pSer^139^] (Upstate Cell Signalling, Billerica, MA) and polyclonal histone H3 antibody (Abcam). Changes in *γ*H2AX levels with respect to histone H3 levels were quantified using ImageJ (Rasband 1997). To inhibit PP2A‐mediated dephosphorylation of *γ*H2AX, 25 nmol/L okadaic acid was added to cells immediately after CPT treatment.

### Comet assay

Neutral comet assays were performed according to protocols described earlier (Olive and Banath 2006; Nowsheen et al. 2012). Briefly, replication‐dependent DNA breaks were induced by addition of 1 *μ*mol/L CPT to cell cultures for 1 h. Cells were harvested immediately after treatment and after growth in drug‐free media for 1, 24, and 48 h. Single cell suspensions in 1% low melt agarose were plated on slides, lysed using neutral lysis buffer and electrophoresed for 30 min. Slides were fixed in 70% ethanol and stained with SYBR Gold (Life Technologies). Images were acquired on an UltraVIEW VoX Life Cell Imager (Perkin Elmer, Waltham, MA) equipped with a Zeiss Axiovert 200 and a 20x/0.8 M27 Plan‐Apochromat objective using Volocity software. At least 50 comets were imaged for three independent cell lines for a minimum of 150 comets at each time point. The mean of the tail DNA percentage was calculated using OpenComet (Gyori et al. 2014).

## Results

Skin fibroblast cell lines established from persons with Bloom syndrome (e.g., GM08505) exhibit a spontaneous, approximately 10‐fold increase in the frequency of SCEs compared to cells from unaffected persons; they also present with quadriradial chromosomes in metaphase spreads, are hypersensitive to HU, and show a delay in activating the DNA damage response after exposure to the topoisomerase poison CPT. To determine the ability of eight SNPs that cause single amino acid changes in BLM (Table 1) to complement the cellular defects of Bloom syndrome cells, the SNPs were introduced into *BLM* cDNA in vector pcDNA3 and expressed in cell line GM08505, in which both *BLM* alleles are inactivated by the *blm* ^*Ash*^ mutation. For each of the eight BLM variants at least three stable cell lines, expressing each BLM variant at levels similar to that of wildtype BLM in a skin fibroblast cell line from an unaffected individual (GM00637), were constructed and analyzed for DNA‐damage sensitivity using a clonogenic (survival) assay (Fig. 1). Expression of wildtype BLM improved survival of Bloom syndrome cells GM08505 in the presence of HU to that of GM00637, whereas expression of P690L, R717T, W803R, Y811C, and G972V caused no improvement (Fig. 1C), suggesting that they eliminate BLM function. Survival of GM08505 cells expressing the R791C, P868L and G1120R variants, however, was not significantly different from wildtype BLM (Fig. 1D).

**Figure 2:**
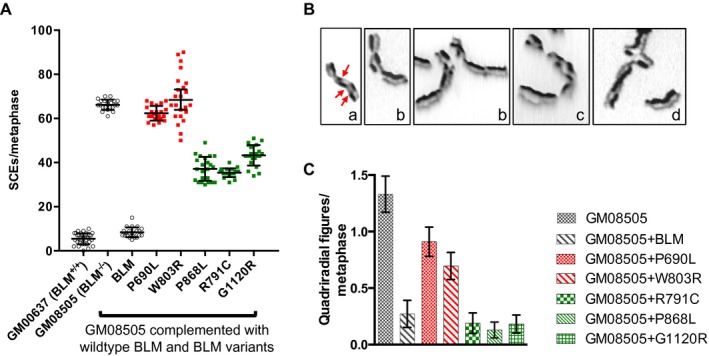
Chromosomal aberrations in GM08505 cells expressing BLM variants. (A) Analysis of sister‐chromatid exchanges (SCEs) by differential sister‐chromatid staining of metaphase chromosomes identifies two groups of BLM variants; variants that cause hypersensitivity to HU (see Fig. 1C), here represented by P690L and W803R, show SCE levels similar to GM08505, whereas variants that do not cause hypersensitivity to 0.5 mmol/L HU (R791C, P868L, G1120R) (Fig. 1D) had reduced levels of SCEs compared to Bloom syndrome cells, but levels were still higher than in GM08505 cells expressing wildtype BLM. SCEs per 46 metaphase chromosomes are shown. (B) Types of chromosomal aberrations commonly observed in Bloom syndrome cells include (a) SCEs, (b) chromatid gaps, (c) segmented chromosomes, and (d) quadriradial chromosomes. (C) Appearance of quadriradial chromosomes in metaphase spreads of GM08505 cells expressing BLM variants R791C, P868L, and G1120R is reduced to wildtype levels, whereas expression of P690L or W803R variants caused significantly elevated formation of quadriradial chromosomes.

The decreased ability of BLM‐deficient cells to dissolve intermediates of HR as noncrossovers is thought to be a cause of the increased frequency of exchange of genetic material between sister chromatids, which can be visualized by differential staining with Hoechst/Giemsa after two rounds of cell division in the presence of the thymidine analog BrdU (Perry and Wolff 1974) (Fig. 2B, panel a). We measured a 12‐fold increase in the SCE frequency in GM08505 cells compared to GM00637 (Fig. 2A), consistent with previous findings in cell lines derived from persons with Bloom syndrome (Ellis et al. 1999). Since the P690L, R717T, W803R, Y811C, and G972V mutations caused the same HU hypersensitivity in human cells as the known Bloom syndrome causing blm^*Ash*^ mutation (Fig. 1C), and the same HU hypersensitivity as the helicase‐dead *blm‐K695A* mutation in yeast (Mirzaei and Schmidt 2012) we focused the evaluation of chromosome abnormalities on the R791C, P868L, and G1120R alleles, and only included two representatives from the group of null alleles, P690L (Walker A motif) and W803R (conserved aromatic‐rich loop). As expected, we found that expression of wildtype BLM in GM08505 lowered the SCE frequency to levels seen in normal fibroblasts whereas expression of variants that exhibited a null phenotype in the HU survival assay had no significant effect on SCE frequency, remaining 7‐fold (P690L) to 8‐fold (W803R) higher than cells expressing wildtype BLM (Fig. 2A). Expression of variants R791C, P868L, and G1120R, which suppressed HU hypersensitivity of GM08505 cells as effectively as wildtype BLM, lowered SCE frequency of Bloom syndrome cells (*P *<* *0.01) (Fig. 2A), but levels were still significantly higher (4–5‐fold, *P *<* *0.01) than in cells expressing wildtype BLM, indicating reduced BLM activity.

In contrast with genetic exchange between sister chromatids, recombination between homologous chromosomes can give rise to LOH; it can thus be considered mutagenic and a driver of tumorigenicity in Bloom syndrome. Such recombination events between nonsister chromatids of homologous chromosomes and, potentially, nonhomologous chromosomes are captured in metaphase spreads as quadriradial chromosomes (Fig. 2B, panel d). As expected, we did not find quadriradials in normal GM00637 fibroblasts, but observed ~13 for every 10 metaphases in GM08505 cells (Fig. 2C). Quadriradial formation was reduced ~5‐fold to fewer than three quadriradials for every 10 metaphases upon expression of wildtype BLM. The same reduction was achieved by expression of R791C, P868L, and G1120R, whereas quadriradials still formed readily in GM08505 cells expressing P690L or W803R (Fig. 2C).

**Figure 3:**
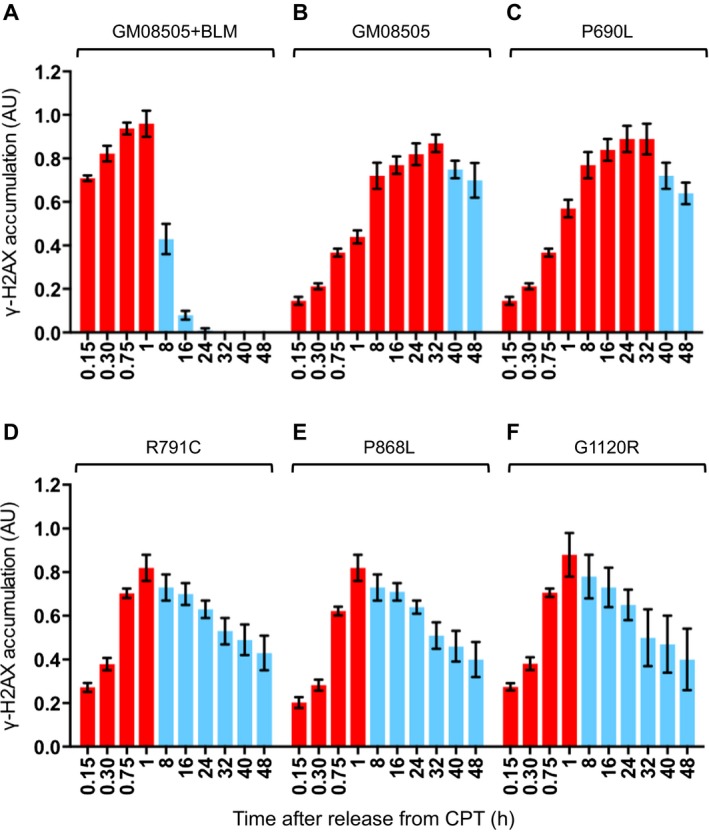
Response of Bloom syndrome cells (GM08505) expressing BLM variants to replication‐dependent DNA breaks. DNA breaks were induced by exposure to 1 *μ*mol/L CPT for 1 h. Accumulation and elimination *γ*H2AX in comparison to histone H3 was quantified by Western blot of extracts from cells collected over a 48 h time course (Fig. S1). Increasing levels of *γ*H2AX are indicated in red, and declining levels in blue. Three independent, stable cell lines were analyzed for GM08505 cells expressing wildtype BLM (A) or BLM variants (C–F). Mean ± SD is shown.

In addition to increased recombination events between sister and nonsister chromatids, cells from Bloom syndrome patients show a delay in the induction of the DNA damage response after exposure to genotoxic agents, such as CPT (Rao et al. 2005), which at low concentrations (≤1 *μ*mol/L) induces replication‐specific DSBs (Holm et al. 1989; Pommier 2006). Phosphorylation of the histone H2A variant H2AX at serine 139 is one of the first markers of DSB formation in eukaryotic cells (Rogakou et al. 1998). Phosphorylated H2AX, *γ*H2AX, is thought to recruit and retain DNA damage response and repair factors at DSBs and increase their accessibility to the DSB site by facilitating chromatin remodeling (Celeste et al. 2002; Fernandez‐Capetillo et al. 2002; Stucki et al. 2005; Tsukuda et al. 2005; Kolas et al. 2007). Results obtained from an H2AX‐S139A mutant suggest that H2AX phosphorylation also plays a role in checkpoint activation (Rios‐Doria et al. 2009). Upon completion of DSB repair, elimination of *γ*H2AX, either by dephosphorylation or exchange with H2AX, is needed for cell cycle progression (Rogakou et al. 1998; Chowdhury et al. 2005; Keogh et al. 2006). To determine the effect of BLM variants on the induction of the DNA damage response we exposed cells to 1 *μ*mol/L CPT for 1 h, removed the drug, and assessed the accumulation of *γ*H2AX every 10 min for 1 h (Fig. 3). To follow completion of DSB repair and cessation of the DNA damage response we extended this time course to 48 h to evaluate the kinetics of the elimination of cellular *γ*H2AX. *γ*H2AX accumulation in Bloom syndrome cells complemented with wildtype BLM peaked 1 h after CPT removal and then *γ*H2AX levels declined rapidly, being undetectable by Western blot after 24 h (Fig. 3A, Fig. S1). In contrast, in Bloom syndrome cells *γ*H2AX levels did not peak until 32 h after drug removal and declined only slowly, with most of the H2AX still phosphorylated after 48 h (Fig. 3B). Bloom syndrome cells expressing the P690L variant, a representative of the five alleles that were hypersensitive to HU in human cells and in yeast, followed the same kinetics of *γ*H2AX accumulation and elimination as Bloom syndrome cells, further supporting the null phenotype of this BLM variant (Fig. 3C). Interestingly, in Bloom syndrome cells expressing R791C, P868L, or G1120R mutations, *γ*H2AX accumulation reached its peak 1 h after CPT removal as in cells expressing wildtype BLM, but there was distinctly less *γ*H2AX, especially at early time points. Even more noticeable, at the 24‐hour time point, when *γ*H2AX was undetectable in complemented Bloom syndrome cells, most of the *γ*H2AX was still present in these mutants, thus resembling the severely delayed elimination of *γ*H2AX in Bloom syndrome cells (Fig. 3D–F, Fig. S1).

**Figure 4:**
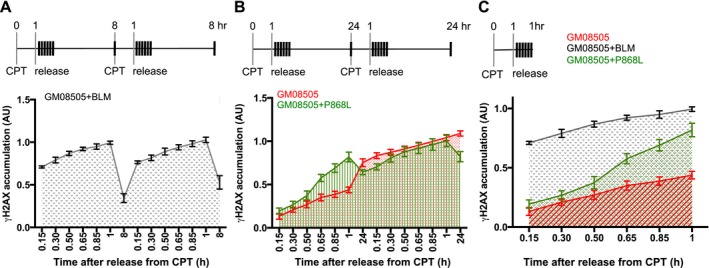
DNA‐damage response after repeated induction of DNA breaks by camptothecin (CPT). (A) GM08505 + BLM cells were exposed to 1 *μ*mol/L CPT for 1 h, released into drug‐free media, and *γ*H2AX levels analyzed by Western blot every 10 min for 1 h and again after 8 h. At the 8‐hour time point cells were exposed to a second round of 1 *μ*mol/L CPT treatment since at this point *γ*H2AX levels in GM08505 + BLM cells had significantly declined (see Fig. 3A). *γ*H2AX levels were analyzed again every 10 min for 1 h and then after 8 h. (B) Cells expressing variant P868L were exposed twice to CPT as in (A), but the second exposure to CPT occurred after 24 h since this was the first time point at which a significant decline in *γ*H2AX was detected in cells expressing the P868L variant (see Fig. 3E). (C) Comparison of *γ*H2AX accumulation in the first hour after CPT removal. *γ*H2AX levels were quantified by Western blot for GM08505 cells and three independent stable cell lines expressing wildtype BLM or the P868L variant. Western blots were analyzed using ImageJ (Schneider et al. 2012). Mean ± SD is shown.

We therefore wanted to test if this impaired elimination of *γ*H2AX in cells with the intermediate phenotype (Fig. 3D–F) affected the cells' ability to mount a response to repeated exposure to a genotoxin. We first tested Bloom syndrome cells complemented with wildtype BLM. Cells were exposed to 1 *μ*mol/L CPT for 1 h and then released into media without CPT, with aliquots being removed every 10 min for 1 h and again after 8 h since at this time point *γ*H2AX levels had declined significantly (Fig. 3A). At this 8 h time point cells were again exposed to 1 *μ*mol/L CPT for 1 h, released into fresh media, and aliquots collected at the same time points as in the first cycle (Fig. 4A). The kinetics of the two cycles of H2AX accumulation/elimination were nearly indistinguishable, suggesting that wildtype cells were capable of mounting proficient responses to repeated insults from CPT. To test blm‐P868L, the intermediate BLM variant with the highest allele frequency in the human population (5.13%), we exposed cells to a second dose of CPT after 24 h (instead of 8 h) because only then did we start to see significant elimination of *γ*H2AX (Fig. 3E). As wildtype cells, blm‐P868L cells responded equally well to both exposures of CPT; however, with a marked >16 h delay in *γ*H2AX elimination in both cycles (Fig. 4B). Despite lower initial H2AX phosphorylation and severely delayed dephosphorylation compared to wildtype cells, blm‐P868L cells responded significantly better to repeated CPT exposure than Bloom syndrome cells (Fig. 3 and 4C).

**Figure 5:**
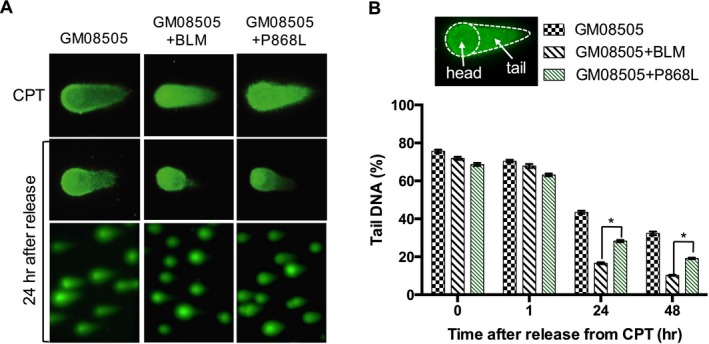
Efficiency of repair of replication‐dependent DNA double‐strand breaks. (A) Neutral comet assay indicates varying levels of double‐strand break repair 24 h after CPT treatment of GM08505 cells expressing no BLM, wildtype BLM, or BLM variant P868L. (B) Tail DNA content was quantified with OpenComet in 150 comets from GM08505 cells and three independent stable cell lines expressing BLM or BLM variant P868L. Mean ± SD is shown. The asterisks indicates *P *<* *0.001.

At low concentrations (<50 nmol/L) okadaic acid selectively inhibits the *γ*H2AX phosphatase PP2A (Honkanen and Golden 2002). Loss of PP2A activity has been shown to cause cell cycle changes, increased sensitivity to DNA damaging agents, and delay in DNA repair, probably enhanced by affecting the activity of other PP2A substrates in addition H2AX, such as ATM, Chk2, and DNA‐PK (Douglas et al. 2001; Dozier et al. 2004; Goodarzi et al. 2004; Chowdhury et al. 2005). Bloom syndrome cells complemented with BLM or blm‐P868L survived treatment with 25 nmol/L okadaic acid with sustained maximum levels of *γ*H2AX (Fig. S2), whereas Bloom syndrome cells died. To test if the marked delay in *γ*H2AX accumulation and elimination after CPT removal in cells expressing P868L corresponded to unrepaired DSBs, we performed a neutral comet assay (Fig. 5). Cells were exposed to 1 *μ*mol/L CPT for 1 h and released into fresh media, with cells being removed for lysis and electrophoresis at the end of the incubation with CPT (0 h) and then after 1, 24, and 48 h. In wildtype cells, DNA breaks were significantly reduced over the 48 h time course, with 23% and 14% of DNA damage remaining after 24 and 48 h respectively. In blm‐P868L cells twice as much DNA damage (28%, *P *<* *0.0001) was still unrepaired after 48 h, indicating a lag of DNA repair of at least 24 h. This presents a significant loss in DNA break repair activity of the P868L variant, but not as great as that of Bloom syndrome cells where the amount of unrepaired DNA breaks after 48 h was three times higher (43%, *P *<* *0.0001) than in wildtype cells.

## Discussion

In this study, we have identified variable functional defects of novel variants of the tumor‐suppressor BLM. Five of the eight variants cause cellular defects that are indistinguishable from those of Bloom syndrome cells, indicating that they encode nonfunctional BLM variants that would cause Bloom syndrome in the homozygous or compound heterozygous state. The amino acid changes in these five BLM variants – P690L, R717T, Y811C, W803R, G972V – cluster along the catalytic cleft between the N‐terminal and the C‐terminal lobe of the ATPase domain of the helicase core. Residues that make up the seven conserved helicase motifs of BLM are located at the surface of this cleft to bind ATP, Mg^2+^ and single‐stranded DNA and, eventually, to hydrolyze ATP. The null phenotype exhibited by the five BLM variants can be explained by the location of the mutated residues in or near these conserved helicase motifs. These new null alleles are the first to inactivate BLM by mutation of residues in or near motifs I (P690L), Ia (R717T), VI (G972V), and the conserved aromatic loop (W803R, Y811C), whereas the known Bloom syndrome‐associated amino acid changes map to motifs 0 (Q672R) and IV (G891E, C901Y) or fall outside of the conserved motifs (I841T, C878R) (Guo et al. 2007). The location of the new missense mutations in the (Walker A) motif I for ATP binding or in other conserved helicase motifs suggests that the null phenotype of the new BLM variants is due to loss of ATPase activity. While it is not surprising that ATPase activity would be required for BLM's role in suppressing SCEs by dissolving dHJ as noncrossovers, our findings also implicate ATPase activity in the role of BLM as a transducer of H2AX phosphorylation in response to replication‐dependent DSBs (Rao et al. 2005), most likely to mediate early events (e.g., resection, unwinding) at collapsed replication forks. Resembling the known Bloom syndrome causing missense mutations, with each having been identified in only one person (German et al. 2007), the new null variants are very rare in the human population, with allele frequencies of <0.01 (Sherry et al. 2001, Exome Variant Server, 2015).

**Figure 6:**
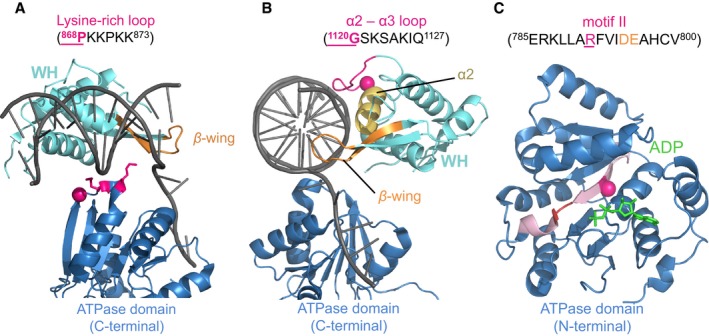
Location in the crystal structure of human BLM of amino acid changes with intermediate functional impact. (A) P868L maps to a lysine‐rich loop at the periphery of the C‐terminal lobe of the ATPase domain. The loop appears to be in contact with dsDNA, which is also contacted by the WH domain. P868L is at the C‐terminal end of a *β*‐strand that transverses lobe 2 of the ATPase domain from the catalytic cleft to the periphery where DNA is contacted, possibly connecting DNA‐substrate binding to ATP‐hydrolysis. The crystal structure of BLM in a complex with ADP and duplex DNA is from PDB (ID:* 4O3M*). (B) G1120 is located in a loop between the second and third *α*‐helix of the WH‐domain that contacts dsDNA. BLM crystal structure is PDB ID *4O3M*. (C) R791 maps to an internal *β*‐strand that precedes the loop of the Walker B motif involved in Mg^2+^‐binding and ATP hydrolysis. Since the loop in helicase motif II (Walker B) is not resolved in *4O3M* (Swan et al. 2014), the R791C mutation is shown in BLM crystal structure *4CDG* (Newman et al. 2013). The amino acid residues changed by the missense mutations are shown as red spheres. Images were generated with PyMOL v1.3.

In addition to new null alleles, we identified the first BLM variant, P868L (rs11852361), that exhibits some, but not all, defects of Bloom syndrome causing mutations and does not, based on its relatively high allele frequency, cause Bloom syndrome. The frequency of P868L in the human population is 5.13%, ranging from 0.6% in the Japanese to 12.3–15.3% (15/122 alleles, 1000 Genomes Project Consortium; 15/98 alleles, CSHL‐HAPMAP) in the population of African descent in the South Western United States (ASW) (International HapMap, 2003, Thorisson et al. 2005, 1000 Genomes Project Consortium, 2012). Homozygosity for P868L in the ASW population was 1 in 61 (1/61 genomes, 1000GENOMES) and 1 in 25 (2/49 genomes, CSHL‐HAPMAP), with the next highest frequencies in the Finnish (3/93 genomes) and the British (2/89 genomes) (International HapMap Consortium 2003, Thorisson et al. 2005, 1000 Genomes Project Consortium 2012). In our study, cells expressing the P868L variant exhibited a significantly elevated SCE frequency (Fig. 2A), an aberrant (slow) DNA‐damage response, and inefficient repair of DNA‐damage‐induced, replication‐dependent DSBs (Fig. 3B). On the other hand, blm‐P868L expressing cells showed similar HU sensitivity and frequency of quadriradial chromosomes as cells expressing wildtype BLM. When mapped onto the recently released crystal structure of human BLM (Swan et al. 2014), P868L affects a residue in a loop of unknown function in the C‐terminal lobe of the ATPase domain. This lysine‐rich loop is not near the catalytic cleft where the null mutations map, but instead appears to contact double‐stranded (ds) DNA bound by the WH domain just before it is unwound (Fig. 6A). Based on this location we propose that replacing the helix breaker proline with hydrophobic leucine disrupts the flexibility and hydrophilicity of the loop, weakening dsDNA binding. Biochemical analysis of the purified BLM variant will be necessary to determine if less efficient dsDNA binding, in and of itself, causes the slower and abnormal DNA break repair exhibited by the P868L variant, or if it is due to less efficient ATP hydrolysis since the ATPase activity of BLM is single‐stranded (ss) DNA‐dependent.

In addition to P868L we identified two other BLM variants that fall into this new class of intermediate (hypomorphic) mutations. G1120R is the first BLM missense mutation from the human population that maps to the WH domain and impairs BLM function. The WH domain is conserved structurally, but not at the amino acid level. We found that one of the few exceptions is the first glycine (G1120) in the loop between the second and third *α*‐helix of the domain (*α*2–*α*3 loop), which is conserved in members of the RecQ helicase family from bacteria, to yeast, to humans. It is still unclear how the WH domain contributes to BLM function since BLM lacking this domain still possesses helicase activity (Gyimesi et al. 2012). Nonetheless, the loop with G1120 has been shown to expand in the related human RecQ helicase WRN upon DNA binding, exposing an arginine for dsDNA binding (Kitano et al. 2010). Similarly, the corresponding loop in the recently released crystal structure of human BLM is near dsDNA (Swan et al. 2014) (Fig. 6 B). We propose that the G1120R mutation, by removing the helix‐terminating glycine, extends the *α*2‐helix, eliminating or at least reducing the loop structure for dsDNA binding. Unlike P868L, G1120R is rare, having been identified once in 2197 genomes (Exome Variant Server 2015).

The third hypomorphic BLM variant that causes elevated SCEs and inefficient DNA break repair, but normal HU sensitivity and wildtype frequency of quadriradial chromosomes, is R791C. The affected residue is in the N‐terminal lobe of the ATPase domain in the internal *β*‐sheet that precedes helicase motif II (Walker B) required for Mg^2+^ binding and ATP hydrolysis (Fig. 6C). Hence, the DNA break repair defect of cells expressing the R791C variant is likely due to diminished ATPase activity. The allele has been identified once in 97 genomes from the Han Chinese in Beijing (1000 Genomes Project Consortium 2012) and twice in 6496 genomes from European‐American and African‐American populations in the United States (Exome Variant Server 2015). Although R791C is so rare it is listed three times in The Cancer Genome Atlas (TCGA); twice in nonsmall cell lung carcinoma and once in a lymphoid neoplasm (Barretina et al. 2012), raising the possibility that it contributes to tumorigenesis.

Taken together, we have identified five new BLM missense mutations that exhibit defects of the Bloom syndrome associated *blm* ^*ASH*^ null allele and are therefore new candidates for Bloom syndrome causing alleles, and three new BLM missense mutations that exhibit some defects (elevated interchromatid crossover recombination (SCEs), slower accumulation and markedly delayed elimination of *γ*H2AX after genotoxin exposure, and inefficient repair of replication‐dependent DSBs), but not others (HU hypersensitivity, accumulation of quadriradial chromosomes) (Table 2). H2AX and its phosphorylation at S139 have been shown to play a role in DSBR by HR between sister chromatids, which is potentially error‐free (Kadyk and Hartwell 1992; Johnson and Jasin 2000), while preventing error‐prone HR, such as single‐strand annealing (SSA) (Xie et al. 2004). Thus, the severe delay in *γ*H2AX accumulation in cells expressing BLM null alleles (e.g. P690L, R717T, W803R, Y811C, and G972V in this study), and the lesser, but significant, delay in cells expressing the P868L, G1120R, or R791C variants may be indicative of dysregulation of DSBR, resulting in increased utilization of SSA or HR between homologs, which are prone to deletions and LOH respectively. In contrast with H2AX phosphorylation and its effects on DSBR, the signaling of *γ*H2AX elimination and the consequences of persistence of *γ*H2AX, as seen in cells expressing fully or partially defective BLM variants in this study, are less well‐understood, but involves direct dephosphorylation by PP2A‐B56*ε* (Xie et al. 2004; Chowdhury et al. 2005). Our findings indicate that the requirement of BLM for the accumulation of *γ*H2AX (Rao et al. 2005) extends to its enzymatic activity, suggesting its requirement for DNA processing rather than protein binding or recruitment at DSBs. Our findings further extend the role of BLM's enzymatic activity to the elimination of *γ*H2AX. Our assays cannot distinguish whether BLM is part of the signaling pathway for *γ*H2AX elimination or the effect is solely through BLM's role in DSB processing and rejoining, but the severely delayed (>40 h) elimination of cellular *γ*H2AX is associated with delayed and, possibly, error‐prone DNA damage repair as well as delays in restoration of chromatin integrity and the cell cycle.

The functional evaluation of new BLM variants in this study also provides insight into the *in silico* predictability of the impact of SNPs on BLM function (Table 1). Initially we used Polyphen (Ramensky et al. 2002) to rank 43 coding SNPs in BLM, with 22 scoring as either probably or possibly damaging. All eight SNPs that are functionally impaired in yeast and in human cells were predicted to be ‘probably damaging’ (score > 2). However, Polyphen was unable to distinguish between the null alleles and the intermediate/hypomorphic alleles. Its successor, PolyPhen2 (Adzhubei et al. 2010), had enhanced predictability, classifying P868L as an intermediate (possibly damaging) allele. In contrast, both SIFT (Kumar et al. 2009) and FIS (Reva et al. 2011) categorized R717T as being ‘tolerated’ or of ‘low’ impact respectively. Thus, for our limited set of variants, this comparison suggests that for the evolutionarily conserved and highly structured catalytic core of BLM, Polyphen2 was the most accurate predictor of functional impact.

The biological relevance of the functional defects of the new class of intermediate BLM variants for human health and aging is currently unclear, as is their importance for a person's sensitivity to genotoxins. Although all defects caused by hypomorphic BLM variants were significantly less pronounced than in Bloom syndrome cells, any increased mutation load over a person's lifetime is likely to be associated with increased cancer risk; this risk is known to be extraordinarily high for *BLM* null alleles, leading to Bloom syndrome, and now remains to be defined for the newly identified class of hypomorphic BLM variants, currently represented by R791C, P868L, and G1120R.

## Conflict of Interest

The authors declare that they have no competing interests.

## Supporting information


**Figure S1.** Representative examples of the response of Bloom syndrome cells (GM08505) expressing wildtype BLM and BLM variants to replication‐dependent DNA breaks induced by exposure to 1 *μ*mol/L CPT for 1 h.
**Figure S2.** Effect of PP2A inhibition on *γ*H2AX accumulation in Bloom syndrome cells expressing wildtype BLM and hypomorphic BLM variants.
