# Supplementary material for: Cellular defects caused by hypomorphic variants of the Bloom syndrome helicase gene BLM
Source: Mol Genet Genomic Med. 2015 Nov 26;4(1):106–19. doi: 10.1002/mgg3.188 (PMC4707026; doi:10.1002/mgg3.188)
Supplement: Supplementary file 1 — Figure S1. Representative examples of the response of Bloom syndrome cells (GM08505) expressing wildtype BLM and BLM variants to replication‐dependent DNA breaks induced by exposure to 1 μmol/L CPT for 1 h. Figure S2. Effect of PP2A inhibition on γH2AX accumulation in Bloom syndrome cells expressing wildtype BLM and hypomorphic BLM variants. [file MGG3-4-106-s001.pdf]

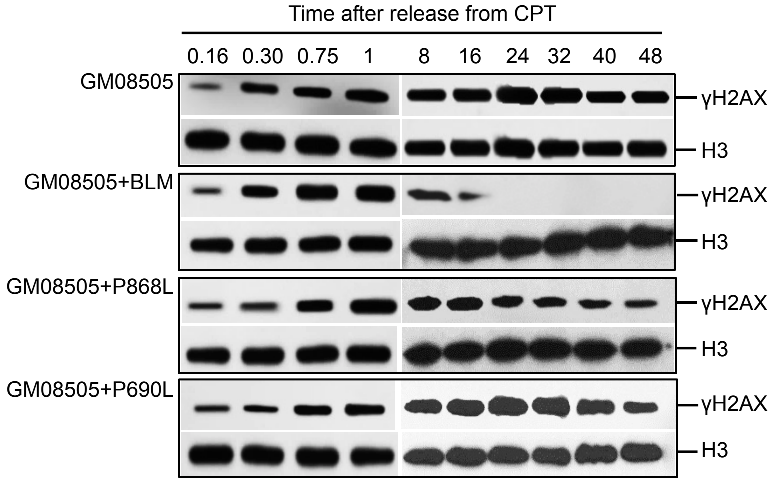

Figure S1. Representative examples of the response of Bloom syndrome cells (GM08505) expressing wildtype BLM and BLM variants to replication-dependent DNA breaks induced by exposure to 1 μM CPT for 1 hour. Accumulation and elimination of γH2AX was determined by Western blot over a 48-hour time course.

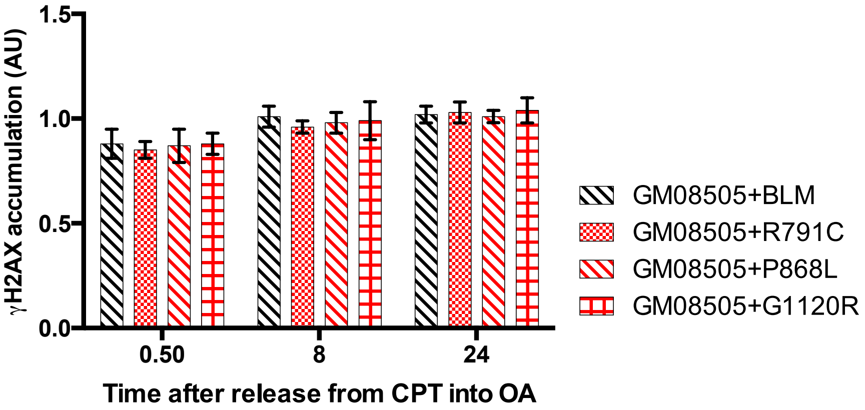

Figure S2. Effect of PP2A inhibition on γH2AX accumulation in Bloom syndrome cells expressing wildtype BLM and hypomorphic BLM variants. Cells were exposed to 1 μM CPT for 1 hour and released into media with 25 nM ocadaic acid (OA). H2AX phosphorylation levels were determined after 0.5, 8 and 24 hours. Non-complemented Bloom syndrome cells were not viable at the 24 hour time point.
